# Medicinal Plants with Anti-*Trichomonas vaginalis* Activity in Iran: A Systematic Review

**Published:** 2019

**Authors:** Hajar ZIAEI HEZARJARIBI, Najmeh NADEALI, Mahdi FAKHAR, Masoud SOOSARAEI

**Affiliations:** 1. Toxoplasmosis Research Center, Department of Parasitology, School of Medicine, Mazandaran University of Medical Sciences, Sari, Iran; 2. Student Research Committee, Department of Parasitology, School of Medicine, Mazandaran University of Medical Sciences, Sari, Iran

**Keywords:** *Trichomonas vaginalis*, Medicinal plants, Systematic review, Iran

## Abstract

**Background:**

Trichomoniasis, due to *Trichomonas vaginalis*, is one of the most common sexually transmitted parasitic diseases in the world such as Iran. This systematic review aimed to explore the studies evaluating the medicinal herbs with anti-*T. vaginalis* activity which used in Iran.

**Methods:**

Articles published in 4 Persian and 4 English databases were obtained between 2000 and 2015 including Google Scholar, PubMed, Science Direct, Scopus, Magiran, Barakatkns (formerly IranMedex), Elm net, and SID (Scientific Information Database). Studies out of Iran, studies on animal models and articles on other parasite species than *T. vaginalis* were excluded from this review.

**Results:**

Twenty-one articles including in vitro experiments, met our eligibility criteria. Thoroughly, 26 types of plants were examined against *T. vaginalis*. Medicinal herbs such as *Artemisia*, *Zataria multiflora*, and *Lavandula angustifolia* are remarkably effective on *T. vaginalis.* As such, use of other parts of these plants in different concentrations and timelines is recommended for future in vivo studies.

**Conclusion:**

The present systematic review provides comprehensive and useful information about Iranian medicinal plants with anti-*T. vaginalis* activity, which would be examined in the future experimental and clinical trials and herbal combination therapy.

## Introduction

*Trichomonas vaginalis(T. vaginalis)* is a flagellated protozoan parasite that attaches to vaginal epithelial cells leading to the occurrence of trichomoniasis. Trichomoniasis is one of the most common sexually transmitted parasitic diseases in the world such as Iran (1). The overall prevalence rate of trichomoniasis in Iran was estimated to be 8% (2). This infection is likely to remain asymptomatic in 10%–50% of the cases. In women, trichomoniasis may lead to different conditions, including vaginitis, dysuria, dyspareunia, premature delivery, premature rupture of membranes, low birth weight, spontaneous abortion, ectopic pregnancy, postpartum endometritis, salpingitis, cervical erosion, chronic cervicitis, cervical cancer, (3, 4) and infertility (5). Although trichomoniasis is associated with no significant clinical symptoms in men, it has been reported to cause urethritis (6).

The risk of *T. vaginalis* increases with the removal of the secretory protease of white blood cells, which protects the vaginal mucosa membrane cells against the human immunodeficiency virus (HIV). Furthermore, some researchers believe *T. vaginalis* to be a co-factor for the transmission of HIV and other sexually transmitted infections (7, 8). Metronidazole is considered the most useful medication in the treatment of trichomoniasis (9). Metronidazole has numerous side effects and in some cases, it has low efficacy in the treatment of infections caused by different bacterial strains. In other words, antibiotic resistance against this medication has increased. Among the main side effects of metronidazole are nausea and vomiting, bad taste in the mouth, gastrointestinal disorders, skin rashes, urticaria, angioedema, dizziness, peripheral neuropathy, and transient neutropenia. There is disagreement among medical specialists regarding the use of this antibiotic during pregnancy, while the use of metronidazole is forbidden during the first trimester of pregnancy (9). Moreover, carcinogenic and mutagenic effects of metronidazole have been reported in animal models (10–12).

WHO has recommended the use of medicinal herbs and natural food compounds for the treatment of various diseases in order to reduce the side effects of chemical drugs (13). Different studies have evaluated the efficacy of medicinal herbs in the treatment of trichomaniasis. In this systematic review, we explored the studies assessing the utilities of medicinal plants with anti-*T. vaginalis* activity in Iran. Evaluation of the studies on the effects of different medicinal herbs on trichomaniasis could provide an overview of the proper treatment of this infection in Iran. The results of this review help researchers find the proper compounds for trichomaniasis treatment with no and or fewer complications.

This study aimed to investigate the effects of anti-*T. vaginalis* medicinal plants in Iran.

## Methods

### Searching plan

**Fig. 1:**
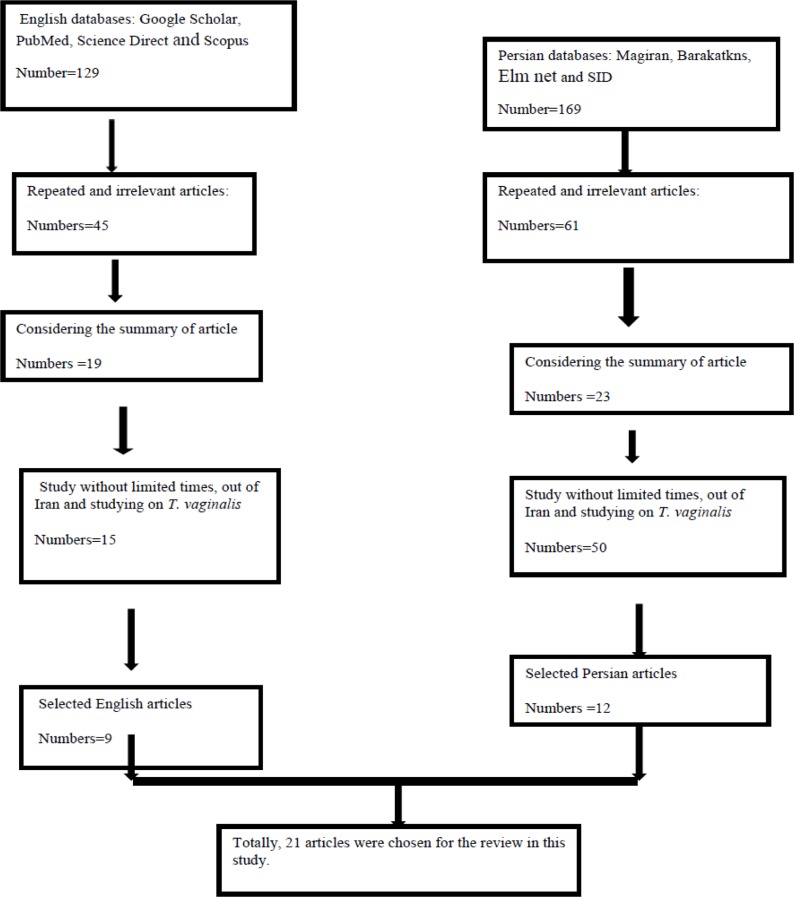
Flowchart describing the study design process

This systematic review was conducted through searching in 8 databases such as Google Scholar, PubMed, Science Direct, Scopus, Magiran, Barakatkns (formerly IranMedex), Elm net, and SID (Scientific Information Database) throughout 2000–2015 (Fig.1). We chose all the articles published in Persian and English languages evaluating anti-*T. vaginalis* medicinal plants using the following keywords: *Trichomonas vaginalis*, trichomoniasis, herb, medicinal plant, herbal medicine, anti-*T. vaginalis* and Iran both separately and combined.

### Quality assessment and article selection

All the articles identified in the aforementioned databases were evaluated by two authors independently. After the review of the titles, abstracts, and full texts of the articles, unrelated studies were excluded from the review. Remaining articles were investigated using quality assessment checklists.

Inclusion criteria of this study were the articles evaluating the in-vitro effects of medicinal herbs on *T. vaginalis* in Iran between 2000 and 2015.

Exclusion criteria were the studies conducted outside the determined deadline, articles on other parasite species than *T. vaginalis*, and invivo studies performed on animal models.

### Data extraction

Essential data including the scientific name of plants, type of herbal extracts, used parts of the plants, extract concentrations, and killing or growth-inhibitory effects were obtained from the selected articles and recorded in prepared forms.

## Results

Overall, 254 articles published from 2000 to 2015 (16 years) in Iran were identified in the literature search. Considering the inclusion and exclusion criteria of the study and after the review of the titles, abstracts, and full texts of these articles, 21 articles were selected for this systematic review. Overall, 26 types of plants had assessed among the selected articles (Table 1).

**Table 1:** Most medicinal plants with anti-*T. vaginalis* activity in Iran

***Scientific name of plant***	***Preparation***	***Plant part***	***Lethal or growth inhibitory***	***Reference***
*Artemisia aucheri*			100% lethal at 0.1, 0.01 and 0.001% concentrations at the beginning of culture.	(14)
*Zataria multiflora*	Essential oils	Aerial parts	100% lethal at 0.1, 0.01, 0.001 and 0.004% concentrations at the beginning of culture.	
*Myrtus communis*			100% lethal at 0.1, 0.01, 0.001 and 0.004% concentrations at the beginning of culture.	
*Artemisia aucheri*			100% lethal at 0.1 and 0.01 mg/mL concentrations respectively	
*Zataria multiflora*	Methanolic	Aerial parts	100% lethal at 0.1 and 0.01 mg/mL concentrations at the beginning of culture	(15)
*Myrtus communis*			100% lethal at 0.1 mg/mL concentration at the beginning of cultivation and 0.01 mg/mL	
*Allium hirtifolium*	Hydroalcoholic Dichloromethanic	Root	MIC for extract of hydro alcoholic and dichloromethanic was 10 and 5 μg/mL respectively	(16)
*Myrtus communis*			IC50 = 0.034 μg/mL	
*Zataria multiflora*			IC50= 0.012 μg/mL	
*Dorstenia barteri*			IC50= 3.2 μg/mL	
*Lavandula angustifolia*			IC50= 0.0015 μg/mL	
*Mentha piperita*			IC50= 0.051 μg/mL	
*Micana cordifolia*	NR	Aerial parts	IC50= 12.5 μg/mL	(17)
*Myrtus communis*	Essential oil Methanolic	Aerial parts	Methanolic extract at concentrations of 0.1 and 0.01 mg/mL and essential oil at concentrations of 0.1, 0.01, 0.001 mg/mL and 0.0004 mg/mL are effective at the beginning of the inoculation and at concentrations of 0.0002 and 0.0001 mg/mL respectively	(19)
*Artemisia absinthium* *Achillea millefolium*	Ethanolic	Leaves	The effects of concentrations of 6.25 to 800 mg/ mL compared to the control group were significant	(20)

IC50: Inhibitory concentration 50%

MIC: The minimum inhibitory concentration

NR: Not reported

*Myrtus communis* and *Zataria multiflora* medicinal plants were reported to have the most anti-*T. vaginalis* activity. Among the studied medicinal herbs, *Z. multiflora* and *Lavandula angustifolia* were observed to apply the most significant inhibitory effects against *T. vaginalis*.

Only a limited number of medicinal plants, such as *Stachys lavandulifolia* and *Eucalyptus camaldulensis*, were found to have minimal inhibitory effects against *T. vaginalis*, and no significant difference was reported between the effects of these plants and control groups.

## Discussion

Quite a lot of evidence based studies have supported the use of herbs in complementary and preventive medicine to facilitate treat of various sickness or disorders. Considering the adverse effects of chemical drugs, increasing antibiotic resistance, and lack of access to many of these medicines, use of medicinal plants could be beneficial in the treatment of infectious diseases.

Many synthetic drugs have a natural origin, such as emetine (34), quinine (anti-malarial drug), and *Artemisinin* (35), as such, parasites of the *Plasmodium* spp. have reported to have no resistance to these agents. In this systematic review, we systematically reviewed the effects of medicinal plants used against *T. vaginalis* in Iran.

Several studies have been conducted in different countries evaluating the potential effectiveness of various medicinal plants against *T. vaginalis*. Some of these herbs include *Garcinia kola*, *Commiphora molmol*, *Persea americana*, *Nigella sativa*, *Eugenia uniflora*, *Polygala decumbens*, *Maytenus imbricata*, *Arbutus unedo*, and *Hypericum polyanthemum,* most of which have beneficial effects against *T. vaginalis* (36–45).

Plants of the genus *Artemisia* exhibit antimicrobial activities in different laboratory animals, while the possible toxic effects of these plants should be evaluated in different cultured cells. In the absence of such toxic effects and efficacy of these medicinal plants in vivo, combinations of these herbs have been used as an appropriate alternative for anti-trichomonial therapy.

Various anti-parasitic and antifungal properties have been attributed to artemisinin, extracted from *Artemisia annua* plant as an important medicinal herb. In this regard, studies were performed to assess the effects of the essence and methanol extract of *Artemisia aucheri (A. aucheri)* on *T. vaginalis*. *A. aucheri* is one of the most important medicinal plants used against *T. vaginalis*, and the essence of this herb had more significant effects compared to the alcoholic extract (14, 15).

Based on the results of the aforementioned studies, the most effective dose of the herbal extract of *A. aucheri* was 0.1 mg/mL (14, 15).

Moreover, plant metabolites containing alkaloids, isoflavonoid glucosides, saponins and sesquiterpene lactones posses anti-*T. vaginalis* activities. (46).

In another study, the antifungal activity of the herbal extracts obtained from the aerial parts of different species of genus *Artemisia* against *Candida albicans*, *Aspergillus flavus*, *A. niger*, *Trichophyton rubrum*, and *Epidermophyton floccosum* were investigated, all of which could be pathogenic to human (47).

The in vitro anti-parasitic effects of *Artemisinin* on *Neospora caninum* were evaluated and it could inhibit the growth of *N. caninum* tachyzoites (48). In this regard, Shuhua et al. also investigated the protective and therapeutic effects of Artemether on animal models infected with *Schistosoma mansoni*. The parasitic infection did not spread in the laboratory animals treated with Artemether during the first month. In addition, the infection decreased by 72%–82% compared to the control group, while treatment replication reduced the infection by 97.2%–100% (49).

Previous studies have reported different antimicrobial and therapeutic properties for pot-herbs such as thyme, *Z. multiflora* and myrtle, *M. communis*. For instance, the anti-*T. vaginalis* properties of the oil and alcoholic extracts of different A*rtemisia* spp. were examined, and the most effective inhibitory dose was determined at 0.1 mg/mL. As such, these extracts could be used as capable anti-*T. vaginalis* agents for in vivo studies in the future (14, 15).

In another in vitro study, inhibitory effects of thyme against *Giardia* cysts were assessed. Concentrations of 1:2, 1:4, 1:10, 1:50, and 1:100 of this herbal extract had significant inhibitory effects against *Giardia* spp. cysts at 30 and 60 min (50).

In this regard, the study was performed to investigate the anti-*T. vaginalis* activity of lavender, *Lavandula angustifolia*. The anti-parasitic effects of the concentrations of 0.1, 0.01, and 0.001 mg/mL of this plant were examined after 3, 4, 5, 6, 12, 24, 48, and 72 h. Concentration of 0.1 of the lavender extract killed all *T. vaginalis* trophozoites after 90 min (8). Lavender plant has been shown to have sedative and antimicrobial properties. The effects of lavender extract on bacteria such as *Streptococcus pneumoniae*, *Streptococcus pyogenes*, *Staphylococcus aureus*, and *Pseudomonas aeruginosa* were evaluated. Minimal inhibitory concentration for *S. pneumoniae*, *S. pyogenes*, *S. aureus*, and *P. aeruginosa* was 0.097, 0.097, 6.25, and 3.125 μg/mL, respectively (51).

In a study, the inhibitory effects of lavender against *A. fumigatus* were evaluated and this plant exerted protective effects against *A. fumigatus* and could be used as a natural anti-fungal agent (52).

The genus *Allium* spp. of the Liliaceae family has more than 600 species, the most important of which are nourishing plants such as garlic (*A. sativum* ) and onion (*A. cepa* ) (34). Several investigations have been performed on different properties of these species (35, 53–56). *A. sativum* has been widely used as a therapeutic plant in the traditional and modern medicine due to its anti-microbial, anti-viral, anti-fungal, and anti-parasitic properties. Furthermore, the results proposed by Moazeni et al. were indicative of the presence of extraordinary *anti-protoscolex* agents in garlic in vitro (57). The findings of Behnia et al. were indicative of the anti-parasitic effects of garlic against *Entamoeba histolytica* denoting no significant difference between the effects of garlic plant and metronidazole in this regard (58). Two species of genus *Allium*, including *A. sativum* and *A. hirtifolium*, were reported to have adequate anti-*T. vaginalis* activity (16). With respect to the in vitro and in vivo effects of garlic extract on flagellated *Giardia lamblia*, this herbal extract had the most significant inhibitory effect against *G. lamblia* at the dose of 80 mg/mL, which resulted in the complete recovery of mice within three days (59).

In addition, the in vitro effects of the liquid extracts of three herbal species of the genus *Allium* (garlic, onion, and shallot) assessed against Giardia *spp.* cysts. Mean of the inhibitory dose of garlic was 107.5±34.1 mg/ mL with the rate of 43.2%. In onion, mean of the inhibitory dose was 102.83±9.88 mg/ mL with the rate of 40.8%, while these rates were determined at 84.66±4.80 mg/mL and 33.6% in shallot, respectively (60,61).

Eucalyptus, *Echinophora platyloba*, is a plant traditionally used in herbal medicine for various conditions. In this regard, Kazemian et al. (21) and Youse et al. (23) investigated the inhibitory effects of this plant against *T. vaginalis.* Methanolic and hydroalcoholic extracts of *E. platyloba* could kill 100% of *T. vaginalis* trophozoites.

According to the findings of the present review, *Mentha longifolia* is one of the most effective medicinal plants against *T. vaginalis* (31). Moreover, *Eucalyptus camaldulensis*, exhibit significant inhibitory effects against *T. vaginalis* and it could be used as safer and effective alternatives of chemical agents in the treatment of this infection in future (21,23,25,26).

## Conclusion

Considering the adverse effects of many current chemical drugs, increasing drug resistance, and lack of access and high costs of the available drugs, it is recommended apply for natural sources and cost-effective medicines in the treatment of parasitic infections (62) such as *T. vaginalis* infection. To date, several natural compounds have been shown to be effectual in the treatment of trichomoniasis. Unfortunately, limited research has been conducted as to investigate the exact effects of all these medicinal plants, and the use of natural anti-*T. vaginalis* agents are not approved by the Food and Drug Administration (FDA).

Various plants including *Artemisia* spp. members of the *Allium* family, potherbs (*Z. multiflora*, *M. communis*), and lavender could exert significant inhibitory effects against *T. vaginalis* and are considered relatively safer and efficient alternatives for treatment of *T. vaginalis* infections rather than metronidazole. The present systematic review provides comprehensive and useful information about Iranian medicinal plants with anti-*T. vaginalis* activity, which would be examined in the future experimental and clinical trials and herbal combination therapy.
